# Physical Activity Capacity Assessment of Patients With Chronic Disease and the 1-Minute Sit to Stand Test: Is There an Interest?

**DOI:** 10.3389/fspor.2022.839509

**Published:** 2022-03-17

**Authors:** Edem Allado, Mathias Poussel, Eliane Albuisson, Jean Paysant, Margaux Temperelli, Oriane Hily, Anthony Moussu, Noura Benhajji, Gerôme Gauchard, Bruno Chenuel

**Affiliations:** ^1^Université de Lorraine, CHRU-Nancy, University Center of Sports Medicine and Adapted Physical Activity, Nancy, France; ^2^Université de Lorraine, DevAH, Nancy, France; ^3^OMEOS, Nancy, France; ^4^CHRU-Nancy, Direction de la Recherche Clinique et de l'Innovation, Nancy, France; ^5^Université de Lorraine, CNRS, IECL, Nancy, France; ^6^Université de Lorraine, Faculté de Médecine, Département du Grand Est de Recherche en Soins Primaires: DEGERESP, Nancy, France; ^7^Université de Lorraine, CHRU-Nancy, Rehabilitation Medicine Department, Nancy, France

**Keywords:** adapted physical activity, chronic disease, 1-minute Sit to Stand test, aerobic capacities, exercise aerobic

## Abstract

**Objective:**

This study aimed to evaluate the efficacy of the 1-minute Sit to Stand test (1MSTS) to determine physical activity capacities for patients with chronic disease.

**Methods:**

For this cross-sectional study, we studied fifty patients with chronic disease and no beta-blocker treatment. They systematically performed a cardiopulmonary exercise test to determine maximal oxygen uptake (V'O_2_max). We considered two groups of patients based on the results of the functional evaluation of exercise performance: (1) No limitation in exercise performance (V'O_2_max greater or equal to 80% of the theoretical reference) and (2) limited exercise performance (V'O_2_max <80% of the theoretical value). All patients also received an 1MSTS on the same day.

**Results:**

We found 22 (44.0%) patients with normal exercise capacity and 28 (56.0%) patients with limited exercise performance. In this sample, mean 1MSTS repetitions were 27.1 (7.1) and 25.2 (8.7), respectively. There were no significant differences between the two groups for repetition and Borg Scale end test. The correlation between V'O_2_max measured during the exercise test and 1MSTS repetitions displayed a positive slope [*r* = 0.401 (95% CI 0.114–625)].

**Conclusion:**

This study demonstrated a moderate relationship between 1MSTS and V'O_2_max for patients with chronic disease. 1MSTS did not permit the precise determination of physical activity capacities in this sample.

## Highlights

- The correlation between the 1-minute Sit to Stand test and V'O_2_max is moderate for patients with chronic disease.
- The 1-minute Sit to Stand test cannot determine the physical activity capacities of patients with chronic disease.

## Introduction

Assessing a patient's ability to engage in an adapted physical activity (APA) is an important issue to ensure safe practice (Pedersen and Saltin, 2015; U.S. Department of Health Human Services, 2018). In 2016, the French Health Ministry authorized the prescription of APA for patients with chronic diseases (Loi n°2016-41, art. L. 144 du 26 Janvier 2016 de Modernisation de Notre Système de Santé, 2016), but assessment of cardiovascular risk and aerobic exercise capacity is also essential (American College of Sports Medicine et al., 2018; Haute Autorité de Santé, 2019). The Sit to Stand test was developed to assess lower limb capability (Csuka and McCarty, 1985). The 1-minute Sit to Stand (1MSTS) test version described by Koufaki et al. in 2002 was developed to assess a patient's aerobic capacity within a short time and in a limited space (Painter et al., 2000; Koufaki et al., 2002; Bohannon and Crouch, 2019; Simonelli et al., 2021). Indeed 1MSTS only requires a chair, stopwatch, and <2 m^2^ of floor space.

Our main goal was to evaluate the level of the relationship between the aerobic exercise performance capacity and 1MSTS results in a sample of patients suffering from chronic disease following a hospital based adapted physical activity program.

## Methods

This is a cross-sectional monocentric study performed at the Nancy University Center of Sports Medicine and APA, France, between February 2020 and July 2021, in patients requiring implementation of APA. APA is a means that allows the movement of people who, due to their physical, mental, or social status, cannot perform physical activity under normal conditions. The inclusion criteria were age >18 years old, with the ability to perform a cardiopulmonary exercise test and participate in supervised APA practice using a telemetry wireless system to monitor and record HR during the first session. The exclusion criteria were beta-blocker treatment and joint pain or limitations.

### Demographic, Clinical Data, and Intervention

Data collected from the included patients were age, gender and body mass index (BMI). We classified the patient's referral department categories behind the APA program into four groups: Obesity, Rheumatology and Others. A maximum cardiopulmonary exercise test was carried out on all included patients.

Following the cardiopulmonary exercise test, the sample was split into two groups (Shvartz and Reibold, 1990; American Thoracic Society American College of Chest Physicians, 2003; Pedersen and Saltin, 2015; Carré, 2016): (1) No limitation of exercise performance capacities: those with normal maximal oxygen uptake [(V'O_2_max) ≥80% of the reference value] and (2) limitation of exercise performance capacities defined by a reduced V'O_2_max (<80% of the reference value). We used Wasserman equation for reference values (Roca et al., 1997; Radtke et al., 2019). The actual peak HR (HRpeak) was measured during the exercise test.

- Exercise test procedure: V'O_2_max was established during an incremental exhaustive exercise test on a cycle ergometer (eBike, GE Healthcare, France). For each patient, a progressively increasing work rate protocol was designed (rest, followed by an incremental phase of exercise every minute) so that volitional exhaustion is reached following 8–12 min of exercise. Respiratory and metabolic variables (minute-ventilation, tidal volume, frequency of breathing, V'O_2_, V'CO_2_) were measured breath-by-breath through a mask connected to a pneumotachograph and O_2_ and CO_2_ analyzers (Vyntus^TM^ CPX, Vyaire, Germany). Criteria for the achievement of V'O_2_max were HR >90% of the maximal reference value HR (210–0.65 × age), respiratory exchange ratio [(RER) = V'CO_2_/V'O_2_] > 1.1 or V'O_2_ plateau (American Thoracic Society American College of Chest Physicians, 2003; Pedersen and Saltin, 2015; Metz et al., 2018).

The 1MSTS was measured before the exercise test on the same day according to international recommendations (Koufaki et al., 2002; Vaidya et al., 2016). The test was performed using a chair of standard height of 45 cm without arm rests over 1 min. The patient had to be seated upright on the chair positioned against a wall. The patient sat with the knees and hips flexed to 90°, feet placed flat on the floor, hip-width apart, and the hands placed on the hips. Every rise from the chair was validated to check if a complete sit-to-stand-to-sit sequence was achieved. The Modified Borg Scale was used to assess fatigue and the number of repetitions was measured. HR before and after 1MSTS was measured.

### Statistical Analysis

Both descriptive and comparative analyses were made by accounting for the nature and distribution of the variables. Qualitative variables were described as frequencies and percentages; quantitative variables were evaluated using the mean ± standard deviation (SD) or with the median and interquartile range (IQR). The chi-square test or Fisher's exact test with, if necessary, the exact calculation of Fisher, was used for the ordinal or nominal data analysis. We used the student *T*-test to compare age, BMI and HR. Binary logistic regression analyses were performed to analyze exercise capacities with age, BMI, gender and Sit to Stand test (number of complete repetitions). We used Pearson's correlation to analyze the relationship between the 1MSTS test and V'O_2_max. For interpreting Pearson's correlation we used this scale: 0.00–0.10 Negligible correlation, 0.10–0.39 Weak correlation, 0.40–0.69 Moderate correlation, 0.70–0.89 Strong correlation, 0.90–1.00 Very strong correlation (Schober et al., 2018). The significance level was set at 0.05 for the entire study. IBM™ SPSS Statistics v23 was used for the data analysis.

### Ethics and Dissemination

All data used were obtained from the medical records. No supplementary examination was necessary for patients to meet the inclusion criteria. This study is registered with the Information Technology and Freedoms Commission for the University Hospital of Nancy (file number: 2021PI191) and on Clinicaltrials.gov (NCT05146544) and was designed in accordance with the general ethical principles outlined in the Declaration of Helsinki. The protocol of this study was approved by the Information Technology and Freedoms Commission. All patients gave their consent for the use of their medical data during the period they received medical care at the University Hospital. The Ethics Committee waived the requirement of written informed consent for participation.

## Results

### Demographic, Clinical Data, and Intervention

Fifty patients were included in the study, 41 (82%) of whom were women. Mean age was 44.1 (±10.9) years with a mean BMI of 36.5 (±9.1) kg/m^2^. We found 28 (56.0%) patients with limited exercise capacities and 22 (44.0%) patients with normal exercise performance. There were no significant differences between the two groups for demographic and clinical characteristics, except for gender (*p* = 0.028) (Table 1). But multivariate analysis corrected this, only age was significant with *p* = 0.021 (*p* = 0.056 for gender).

**Table 1:** Baseline demographic and clinical characteristics (*n* = 50).

	**Lower exercise capacities (*n* = 28)**	**Normal exercise capacities (*n* = 22)**	^*^p-value
Women	20 (71.4)	21 (95.5)	**0.028**
Age, years	41.6 (10.9)	47.3 (10.3)	0.064
Body mass index, kg/m^2^	36.1 (11.1)	37.1 (5.9)	0.685
**Patient referral department categories**			
- Obesity	15 (53.6)	15 (68.2)	0.624
- Rheumatology	5 (17.9)	3 (13.6)	
- Nephrology	1 (3.6)	0 (0.0)	
- Diabetology	0 (0.0)	1 (4.5)	
- Internal Medicine	3 (10.7)	1 (4.5)	
- Other	4 (14.3)	2 (9.1)	
Sit to Stand test: number of complete repetitions	25.2 (8.7)	27.1 (7.1)	0.386
Heart Rate before Sit to Stand test	100.9 (15.2)	99.0 (16.6)	0.687
Heart Rate after Sit to Stand test	135.5 (15.8)	141.3 (23.6)	0.339
Borg Scale after Sit to Stand test	6.3 (2.2)	6.1 (2.0)	0.793

*Data are presented as n (%) for dichotomous variables, mean (SD) for continuous demographic variables with normal distribution and median (interquartile range) with non-normal distribution*.

* *For univariate analyse, we use the student T-test to compare age and body mass index, and chi-square test or Fisher's exact test for other variables*.

### Relationship Between Physical Activity Capacities and Results of 1MSTS

In this sample, for normal and limited exercise patients, the 1MSTS mean repetitions were 27.1 (7.1) and 25.2 (8.7), respectively, with no significant difference (*p* = 0.386). There were no significant differences between the two groups for HR and Borg Scale after the test (*p* = 0.339 and *p* = 0.793, respectively).

**Figure 1:**
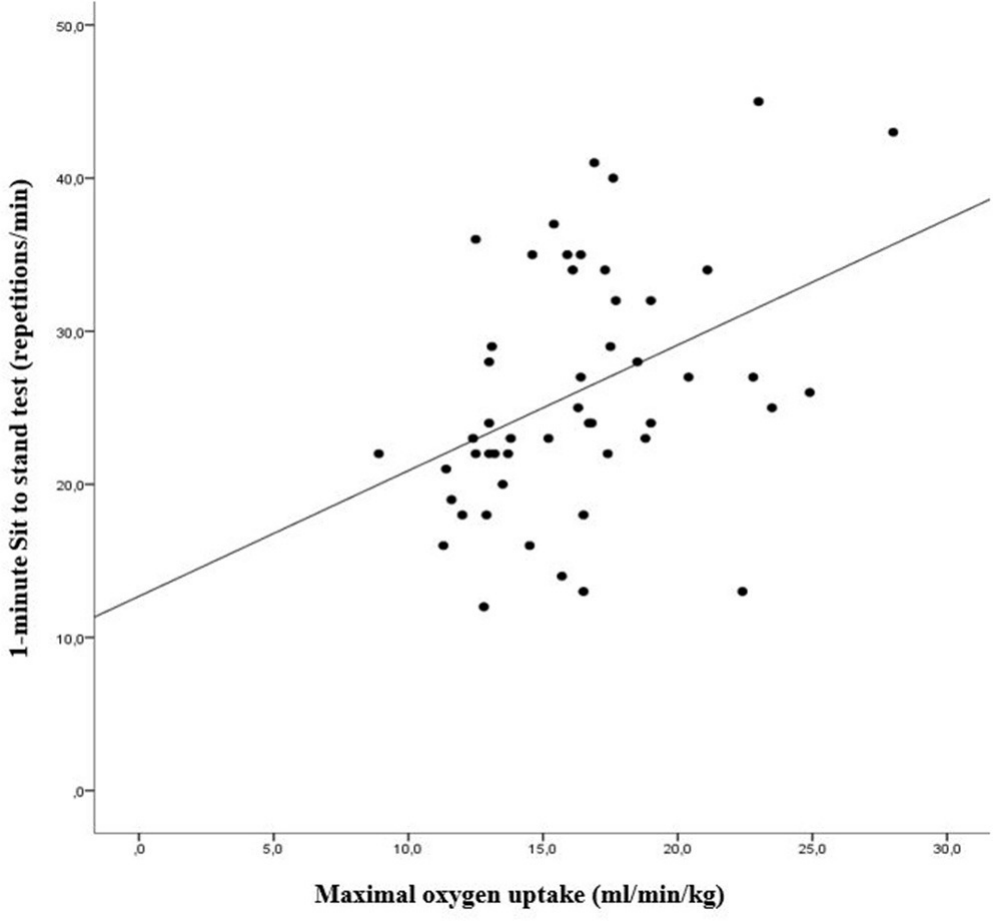
The relationship between matrix maximal oxygen uptake (V'O_2_max) measured during the exercise test and 1-minute Sit to Stand test repetitions (1MSTS) by level exercise capacity (linear regression modeling). Pearson correlation coefficient for all samples: [*r* =0.401 (95% CI 0.114–0.625)] correlation formula: (*y* = 12.69 + 0.82 *x*) with *y* = 1MSTS repetitions and *x* = V'O_2_max) (For normal exercise capacities: [*r* =0.027 (95% CI −0.348 to 0.460)], and for decreased exercise capacities [*r* =0.569 (95% CI 0.194–0.792)].

The correlation between V'O_2_max measured during the exercise test and 1MSTS repetition by exercise performance level showed a positive slope [*r* = 0.401 (95% CI 0.114–625)] (Figure 1).

## Discussion

The study described a moderate correlation between 1MSTS and V'O_2_max in patients suffering from chronic disease following APA. Indeed, it was not possible to ascertain exercise aerobic performance capacity with the 1MSTS. The 1MSTS test was developed in 2002 to assess the aerobic exercise capacities of patients with chronic disease (Koufaki et al., 2002) and was found to be a good alternative to the Six-Minute Walk Test (6MWT) which, unlike the 1MSTS which only needs a small space, requires a corridor at least 30 m long for effective evaluation (Ozalevli et al., 2007; Puhan et al., 2013). Our study revealed a moderate correlation between V'O_2_max and 1MSTS in patients with normal exercise capacities, but the result was better for patients with lower exercise capabilities (Figure 1). Our study contrasts with the previous study made by Nakamura et al. that showed a very strong correlation between V'O_2_max and 1MSTS repetition intervals (*r* = 0.94) unlike our result which based on absolute repetition number (Nakamura et al., 2014). Indeed, Nakamura study was carried out on a population of 10 young people without obesity [age mean: 22.3 (2.2) years and BMI mean 22.5 (4.1) kg/m^2^]. To date, no study has been carried out in such equivalent population. However, similar analyses carried out using the 6MWT show a positive correlation of a similar order of magnitude (0.42–0.58) (Guyatt et al., 1985; Cahalin et al., 1996).

In the case of elite athletes, the V'O_2_max alone cannot explain performance. In this case a moderate correlation between the V'O_2_max and the time to exhaustion (*r* = −0.63). Indeed, the influence of methods and mental state influence performance. On the other hand, in our population, VO_2_max is a faithful reflection of exercise capacities.

The first publication referring to the Sit to Stand test aimed only at assessing the capacity of the lower limb extensor muscles (Csuka and McCarty, 1985). In fact, the initial test consisted of recording the time taken to stand ten times from a standard chair. In 2002, Koufaki et al. standardized the duration of this test to 1 min, to count the number of repetitions required to define the muscular aerobic capacities of patients with respiratory or chronic disease (Koufaki et al., 2002; Ozalevli et al., 2007; Puhan et al., 2013; Bohannon and Crouch, 2019). However, the 1MSTS test has limitations in young patients or in those with scarcely any functional restraints as it reflects the strength of the lower muscles and not of overall aerobic capacity (Ritchie et al., 2005; Rausch-Osthoff et al., 2014; Zanini et al., 2015; Vaidya et al., 2016; Bohannon and Crouch, 2019). Indeed, a strong correlation is observed (*r* = 0.8) in healthy subjects aged 55–70, with no chronic disease, while the association is weaker in patients presenting a Chronic Obstructive Pulmonary Disease (*r* = 0.50). It is probably the reason for the moderate correlation observed in our sample of patients with capacity limitations.

This study has limitations. Firstly, the size sample of only 50 patients from a single hospital center, prevents any extension of the results. In addition, the inclusion of many obese participants limited a subgroup exploration of different chronic pathologies.

The main strength of this study is the focus of V'O_2_max as a gold standard to assess the functional capacities of patients. It also incorporates all the recommendations of the French Health Act issued by the Ministry of Social Affairs and Health.

In conclusion, our study explored the moderate relationship between 1MSTS results and V'O_2_max as well as the possible inability of this test to detect the physiological exercise aerobic capacity of patients with chronic disease. However, this test may be of interest for characterizing an evolution in the general capacities of the patient practicing an adapted physical activity. An analysis of a larger and heterogeneous population and longitudinal clinical trial design would be necessary to make this observation more explicit, and hence pave the way for the implementation of new exercise assessment programs for patients with chronic disease requiring APA.

## Data Availability Statement

The raw data supporting the conclusions of this article will be made available by the authors, without undue reservation.

## Author Contributions

EAll had the original idea. EAll, BC, and EAlb designed and conceived the protocol. EAll and BC drafted the manuscript. EAll, MP, EAlb, JP, MT, OH, AM, NB, GG, and BC critically revised the manuscript for methodology and intellectual content. All authors approved the final version of this manuscript.

## Funding

The sponsor was CHRU de Nancy (Direction de la Recherche et de l'Innovation).

## Conflict of Interest

The authors declare that the research was conducted in the absence of any commercial or financial relationships that could be construed as a potential conflict of interest.

## Publisher's Note

All claims expressed in this article are solely those of the authors and do not necessarily represent those of their affiliated organizations, or those of the publisher, the editors and the reviewers. Any product that may be evaluated in this article, or claim that may be made by its manufacturer, is not guaranteed or endorsed by the publisher.

## Abbreviations

1MSTS: 1-minute Sit to Stand test
6MWT: Six-Minute Walk test
APA: adapted physical activity
BMI: body mass index
HR: heart rate
HRpeak: peak heart rate
IQR: interquartile range
RER: respiratory exchange ratio
SD: standard deviation
V'O_2_max: maximal oxygen uptake.

## References

[B1] American College of Sports Medicine Riebe D. Ehrman J. K. Liguori G. Magal M. (eds.). (2018). ACSM's Guidelines for Exercise Testing and Prescription. 10th Edn. Philadelphia, PA: Wolters Kluwer.

[B2] American Thoracic Society and American College of Chest Physicians (2003). ATS/ACCP Statement on cardiopulmonary exercise testing. Am. J. Respir. Crit. Care Med. 167, 211–277. 10.1164/rccm.167.2.211 12524257

[B3] Bohannon R. W. Crouch R. (2019). 1-minute sit-to-stand test: systematic review of procedures, performance, and clinimetric properties. J. Cardiopulm. Rehabil. Prev. 39, 2–8. 10.1097/HCR.0000000000000336 30489442

[B4] Cahalin L. P. Mathier M. A. Semigran M. J. Dec G. W. DiSalvo T. G. (1996). The six-minute walk test predicts peak oxygen uptake and survival in patients with advanced heart failure. Chest 110, 325–332. 10.1378/chest.110.2.325 8697828

[B5] Carré F. (2016). Analyse de la consommation d'oxygène en pratique cardiologique. La Lettre du Cardiologue, 6.

[B6] Csuka M. McCarty D. J. (1985). Simple method for measurement of lower extremity muscle strength. Am. J. Med. 78, 77–81. 10.1016/0002-9343(85)90465-6 3966492

[B7] Guyatt G. H. Sullivan M. J. Thompson P. J. Fallen E. L. (1985). The 6-minute walk: a new measure of exercise capacity in patients with chronic heart failure. Can. Med. Assoc. J. 132, 5. 3978515 PMC1345899

[B8] Haute Autorité de Santé (2019). Guide de Promotion, Consultation et Prescription Médicale d'Activité Physique et Sportive Pour la Santé Chez les Adultes.

[B9] Koufaki P. Mercer T. H. Naish P. F. (2002). Effects of exercise training on aerobic and functional capacity of end-stage renal disease patients: exercise training respones in ESRD. Clin. Physiol. Funct. Imaging 22, 115–124. 10.1046/j.1365-2281.2002.00405.x 12005153

[B10] Loi n°2016-41, art. L. 144 du 26 Janvier 2016 de Modernisation de Notre Système de Santé (2016).

[B11] Metz L. Thivel D. Peirrera B. Richard R. Julian V. Duclos M. (2018). A new equation based on the 6-min walking test to predict VO _2peak_ in women with obesity. Disabil. Rehabil. 40, 1702–1707. 10.1080/09638288.2017.1304582 28345359

[B12] Nakamura K. Ohira M. Yokokawa Y. (2014). The effect of different standing up frequencies in sit-to-stand exercise on oxygen uptake. J. Phys. Ther. Sci. 26, 1631–1633. 10.1589/jpts.26.1631 25364131 PMC4210416

[B13] Ozalevli S. Ozden A. Itil O. Akkoclu A. (2007). Comparison of the sit-to-stand test with 6min walk test in patients with chronic obstructive pulmonary disease. Respir. Med. 101, 286–293. 10.1016/j.rmed.2006.05.007 16806873

[B14] Painter P. Carlson L. Carey S. Paul S. M. Myll J. (2000). Physical functioning and health-related quality-of-life changes with exercise training in hemodialysis patients. Am. J. Kidney Dis. 35, 482–492. 10.1016/S0272-6386(00)70202-2 10692275

[B15] Pedersen B. K. Saltin B. (2015). Exercise as medicine - evidence for prescribing exercise as therapy in 26 different chronic diseases. Scand. J. Med. Sci. Sports 25, 1–72. 10.1111/sms.12581 26606383

[B16] Puhan M. A. Siebeling L. Zoller M. Muggensturm P. ter Riet G. (2013). Simple functional performance tests and mortality in COPD. Eur. Respir. J. 42, 956–963. 10.1183/09031936.00131612 23520321 PMC3787814

[B17] Radtke T. Vogiatzis I. Urquhart D. S. Laveneziana P. Casaburi R. Hebestreit H. (2019). Standardisation of cardiopulmonary exercise testing in chronic lung diseases: summary of key findings from the ERS task force. Eur. Respir. J. 54, 1901441. 10.1183/13993003.01441-2019 31857385

[B18] Rausch-Osthoff A.-K. Kohler M. Sievi N. A. Clarenbach C. F. van Gestel A. J. (2014). Association between peripheral muscle strength, exercise performance, and physical activity in daily life in patients with chronic obstructive pulmonary disease. Multidiscipl. Respir. Med. 9, 1–7. 10.4081/mrm.2014.372 25013723 PMC4091091

[B19] Ritchie C. Trost S. Brown W. Armit C. (2005). Reliability and validity of physical fitness field tests for adults aged 55 to 70 years. J. Sci. Med. Sport 8, 61–70. 10.1016/S1440-2440(05)80025-8 15887902

[B20] Roca J. Whipp B. J. Agusti A. G. N. Anderson S. D. Casaburi R. Cotes J. E.. (1997). Clinical exercise testing with reference to lung diseases: indications, standardization and interpretation strategies. Eur. Respir. J. 10, 2662–2689. 10.1183/09031936.97.10112662 9426113

[B21] Schober P. Boer C. Schwarte L. A. (2018). Correlation coefficients: appropriate use and interpretation. Anesth. Analg. 126, 1763–1768. 10.1213/ANE.0000000000002864 29481436

[B22] Shvartz E. Reibold R. C. (1990). Aerobic fitness norms for males and females aged 6 to 75 years: a review. Aviat. Space Environ. Med. 61, 3–11. 2405832

[B23] Simonelli C. Paneroni M. Vitacca M. Ambrosino N. (2021). Measures of physical performance in COVID-19 patients: a mapping review. Pulmonology 27, 518–528. 10.1016/j.pulmoe.2021.06.005 34284976 PMC8221906

[B24] U.S. Department of Health and Human Services (2018). 2018 Physical Activity Guidelines Advisory Committee Scientific Report Washington (DC), 779.

[B25] Vaidya T. de Bisschop C. Beaumont M. Ouksel H. Jean V. Dessables F.. (2016). Is the 1-minute sit-to-stand test a good tool for the evaluation of the impact of pulmonary rehabilitation? Determination of the minimal important difference in COPD. Int. J. Chron. Obstruct. Pulm. Dis. 11, 2609–2616. 10.2147/COPD.S115439 27799759 PMC5079690

[B26] Zanini A. Aiello M. Cherubino F. Zampogna E. Chetta A. Azzola A.. (2015). The one repetition maximum test and the sit-to-stand test in the assessment of a specific pulmonary rehabilitation program on peripheral muscle strength in COPD patients. Int. J. Chron. Obstruct. Pulm. Dis. 10, 2423–2430. 10.2147/COPD.S91176 26648705 PMC4648595

